# Navigating neurosurgical care in low- and middle-income countries from Africa: a narrative review of barriers and facilitators

**DOI:** 10.1097/MS9.0000000000003844

**Published:** 2025-09-02

**Authors:** Alex Mwangi Kihunyu, Sjaak Pouwels, Ademola Adeyeye, Ghulam Husain Abbas, Annemarie W. Oldenbeuving, Dharmanand Ramnarain

**Affiliations:** a College of Health Sciences, University of Nairobi, Nairobi, Kenya; b Department of Surgery, Bielefeld University – Campus Detmold, Klinikum Lippe, Detmold, NRW, Germany; c Department of Intensive Care Medicine, Elisabeth-Tweesteden Hospital, Tilburg, Netherlands; d Faculty of Life Sciences and Medicine, King’s College London, London, United Kingdom; e College of Medicine and Health Sciences, Afe Babalola University, Ekiti, Nigeria; f Faculty of Medicine, Ala-Too International University, Bishkek, Kyrgyz Republic; g Department of Medicine, Mass General Brigham, Boston, MA, USA; h Department of Intensive Care Medicine, Saxenburgh Hospital, Hardenberg, Netherlands

**Keywords:** Africa, barriers, facilitators, low- and middle-income countries (LMICs), neurosurgical care

## Abstract

**Background:**

Neurosurgical care is vital for conditions such as traumatic brain injury, brain tumors, and hydrocephalus. In African low- and middle-income countries (LMICs), access remains limited due to systemic and structural challenges. Despite bearing 15% of the global neurosurgical burden, the region faces a massive workforce deficit and underdeveloped infrastructure.

**Objective:**

To synthesize existing evidence on barriers impeding and facilitators improving access to neurosurgical care in African LMICs.

**Methods:**

A narrative review was conducted through a targeted search of PubMed, African Journals Online (AJOL), and Google Scholar for English-language studies published between 2014 and 2024. Studies were included if they focused on African LMICs and addressed access-related barriers or facilitators. In total, 48 studies were selected and analyzed thematically.

**Results:**

Barriers to access include an acute workforce shortage (0.15 neurosurgeons per million people), with only 32 of 54 countries offering neurosurgical training. Infrastructure deficits, such as a lack of Intensive Care Units beds, operating microscopes, and anesthesia services, compound these challenges. Financial constraints are significant, with some families in Eastern Africa paying over 130% of their annual income for surgery. Long travel distances (averaging 144 km), cultural stigma, reliance on traditional healers, and political instability further restrict access. Conversely, facilitators include successful international collaborations (e.g., Duke East Africa Neurosurgery Program, WFNS Rabat Training Centre), adoption of telemedicine and teleradiology, and growth in local research capacity, including disease registries and training exchanges.

**Conclusion:**

Transforming neurosurgical access in African LMICs demands bold investment in rural services, specialist training, and full integration into national health financing.

## Introduction

Neurosurgery is a specialized medical field focused on the surgical management of conditions affecting the brain and spinal cord, including traumatic brain injuries (TBIs), congenital abnormalities, tumors, and infections of the nervous system^[1]^. In 2015, the Lancet Commission on Global Surgery highlighted the global burden of surgical diseases, identifying significant gaps in the accessibility of safe and affordable surgical care^[2]^. Annually, neurological disorders or injuries requiring neurosurgical expertise affect approximately 22.6 million people, with 13.8 million needing surgery^[3]^. Currently, there is an estimated global shortfall of 5.2 million neurosurgical cases, necessitating an additional 22 626 neurosurgeons to bridge this gap^[3]^. Notably, three World Health Organization (WHO) regions, including the Americas (limited to the US and Canada), Europe, and the Western Pacific, have not experienced a workforce shortage^[3]^.

**HIGHLIGHTS**

- A 1700% increase in the neurosurgical workforce is needed in African LMICs to meet current demand.
- Key barriers include severe workforce shortages, limited neurosurgical training programs, infrastructure deficits, financial constraints, and geographic inaccessibility.
- Cultural stigma and political instability further hinder timely access to neurosurgical care in several regions.
- Facilitators such as telemedicine, international collaborations (e.g., Duke East Africa Neurosurgery Project), and local research initiatives show promise in improving access.
- Policy recommendations emphasize multisectoral engagement, dedicated neurosurgical funding, and the establishment of regional hubs under the African Union Health Strategy.

In Africa, neurosurgical cases encompass a wide range of conditions, with TBI being one of the most common due to high rates of road traffic accidents and interpersonal violence. Hydrocephalus remains a significant pediatric concern, often linked to congenital abnormalities and post-infectious complications. Neuro-oncology presents another major challenge, as delayed diagnoses and limited access to advanced imaging and treatment hinder patient outcomes. Other prevalent conditions include congenital malformations, such as spina bifida and encephaloceles, which require specialized surgical interventions but are often inadequately managed due to resource constraints. The high burden of these conditions underscores the urgent need for targeted strategies to improve neurosurgical capacity and access across the continent^[1,3]^.

The African continent bears a significant burden of neurosurgical diseases while facing a critical shortage of specialized medical resources^[4]^. Africa accounts for approximately 15% of the global neurosurgical disease load^[3]^, with low- and middle-income countries (LMICs) representing 82% of cases worldwide^[3]^. Within Africa, around 1.8 million neurological cases go untreated due to a lack of skilled neurosurgeons, necessitating a 1700% increase in the neurosurgical workforce to meet growing demand^[3]^. Moreover, most African LMICs lack access to neurosurgeons, a challenge that persists despite geographical, social, or financial barriers^[3]^.

The disparity in neurosurgical care can be attributed to several factors, including the uneven distribution of neurosurgeons, concentrated in high-income nations like the United States. The migration of skilled healthcare workers, including neurosurgeons, significantly contributes to the workforce shortage across sub-Saharan Africa. More than 5300 physicians from the region are currently practicing in the United States, representing over 6% of the active physician workforce in sub-Saharan Africa, with most originating from Nigeria, South Africa, and Ghana. This reflects broader systemic challenges, where limited local opportunities, underfunded health systems, and security concerns drive the emigration of highly trained professionals, making long-term workforce retention increasingly difficult in many African countries^[5,6]^. In Africa, the ratio of neurosurgeons to patients stands at 1 per 687 740 individuals, far below the WHO’s recommended ratio of 1 neurosurgeon per 200 000 people^[7]^. To illustrate further, 17 LMICs in Africa have between 10 and 40 neurosurgeons, 28 countries have only 1–10 neurosurgeons, and 4 nations have none at all^[7]^. Additional barriers include inadequate resources, such as operating theaters, Intensive Care Units (ICUs), diagnostic equipment (e.g., computed tomography (CT) and magnetic resonance imaging scanners), anesthesia and nursing support, long waiting times, and insufficient training infrastructure^[8]^. A further contributing factor to the limited pool of future neurosurgeons in African LMICs is the limited exposure of medical students to neurosurgical training^[9]^.

Despite these challenges, recent progress has been made in enhancing neurosurgical care. Notably, Africa has seen advancements in surgical technology and an increase in international collaborations, both of which have improved neurosurgical care across the continent^[10]^. Additionally, the expansion of local neurosurgical residency training centers and the establishment of the World Federation of Neurosurgical Societies (WFNS) reference centers provide comprehensive training programs that will ultimately increase the number of neurosurgeons in these LMICs^[11]^.

Successful models from other regions offer valuable insights for strengthening existing facilitators and overcoming barriers. For instance, the Neurosurgery Education and Development model in East Africa has demonstrated the potential of structured international collaborations to enhance local neurosurgical capacity through long-term training partnerships and resource support^[1]^. Similarly, the Lancet Commission on Global Surgery has emphasized the importance of integrated surgical workforce development and infrastructural investments to bridge gaps in essential surgical care worldwide^[2]^.

Latin American countries have made progress in expanding neurosurgical services by increasing training opportunities and decentralizing care to improve access in underserved regions, providing a potential framework for similar initiatives in Africa^[3]^. While cultural, economic, and geographical contexts differ between Latin America and sub-Saharan Africa, core elements of these models, such as regional training hubs, task-sharing, and rural outreach, can be adapted if thoughtfully contextualized. Such cross-regional comparisons yield valuable insights for addressing specialist shortages and enhancing service delivery in resource-limited settings^[12]^. Additionally, efforts in regions like South Asia have shown that strategic policymaking, combined with local and international collaboration, can significantly improve neurosurgical accessibility^[4]^. Implementing these strategies, alongside fostering sustainable neurosurgical training programs and increasing investment in healthcare infrastructure, is essential for enhancing neurosurgical care in Africa^[7]^.

Addressing disparities in neurosurgical care within LMICs requires a comprehensive approach that encompasses capacity building, infrastructural development, and policy advocacy^[13]^. International organizations such as the WFNS and the Lancet Commission on Global Surgery stress the need for greater investment in neurosurgical care in LMICs to meet rising demand and mitigate the global burden of neurosurgical diseases^[11]^.

This study aims to explore the factors that hinder access to neurosurgical care in LMICs across Africa. Furthermore, it seeks to identify the key facilitating factors that have been employed to improve access. Ultimately, this research aims to enhance understanding of how to optimize neurosurgical care in resource-limited settings, thereby improving both patient outcomes and the overall quality of healthcare.

To ensure methodological transparency, this review adheres to the TITAN 2025 Guidelines for ethical and responsible reporting of artificial intelligence use in biomedical research and scholarly work^[14]^.

## Approach to literature selection

This study employed a narrative review process. A targeted search was conducted using PubMed, AJOL (African Journals Online), and Google Scholar to identify studies relevant to key thematic areas of neurosurgical care in LMICs in Africa. Initially, these databases were searched to develop key terms related to the study, followed by a more in-depth exploration. The search terms included combinations such as “Neurosurgical care” AND “Low Middle-Income countries,” “Access to Neurosurgical care” AND “Africa,” “Challenges” affecting “Neurosurgical care in Africa,” “Facilitators” to “Neurosurgical care” AND “Africa,” and “Factors” affecting “Neurosurgical care” in “Africa.” This search strategy yielded 961 documents, from which duplicates were removed.

Articles were included if they met the following criteria: they discussed challenges or facilitators related to accessing quality neurosurgical care, were published in English, focused on Africa, and were published between 2014 and 2024 to ensure the inclusion of the most recent and relevant studies. Exclusion criteria included studies conducted in high-income countries, research conducted outside Africa, articles published in languages other than English, and non-peer-reviewed content such as editorials or opinion pieces. The authors reviewed all titles and abstracts for eligibility, followed by a full-text assessment. After applying these criteria, 48 studies were included in the final synthesis.

Data from the selected studies were organized and reviewed to identify recurring patterns related to neurosurgical care in African LMICs. Using an interpretive narrative approach, the authors examined each article in depth, noting common observations, contextual factors, and thematic insights. The analysis was broadly informed by two overarching domains: barriers to neurosurgical care and facilitators of improved access, with emerging themes within these categories identified organically through repeated reading and reflection.

Themes such as “long travel distances,” “financial burden,” “telemedicine,” “infrastructure gaps,” and “international partnerships” frequently appeared across studies. These thematic insights were not predefined but developed progressively through immersion in the literature. The authors grouped similar findings and synthesized them narratively to explore interconnections, highlight shared challenges, and reflect on practical solutions discussed in the literature. This flexible, iterative process allowed the reviewers to capture both the diversity and convergence of perspectives on access to neurosurgical care across different African contexts.

## Thematic areas of focus

### Barriers to accessing neurosurgical care in LMICs of Africa

#### Workforce deficit

The critical shortage of neurosurgeons in Africa is a major barrier to accessing neurosurgical care, with some countries having only one neurosurgeon for every 687 740 people, far below the WHO-recommended ratio of 1 per 200 000. This shortage is exacerbated by an uneven geographic distribution, with most specialists concentrated in urban centers, leaving rural areas largely underserved.

#### Barriers to neurosurgical education and training programs

Despite having 54 countries, only 32 African nations offer neurosurgical residency programs, many of which face significant challenges such as poor infrastructure, limited funding, and inconsistencies in training curricula. The COVID-19 pandemic further disrupted neurosurgical education, with only 43.9% of training programs in Africa transitioning to online platforms, compared to 98.5% in high-income countries.

#### Resource and infrastructure deficits

Many neurosurgical units in Africa lack essential medical equipment, including operating microscopes, intraoperative fluoroscopy, and ICU beds, which significantly limits the quality of care. Additionally, shortages of trained anesthetists, nurses, and laboratory reagents further delay surgeries and increase postoperative complications.

#### Financial constraints and out-of-pocket costs

The high costs of neurosurgical care pose significant financial barriers, with families in Eastern Africa paying up to 132% of their annual income for procedures, not including additional costs for transportation and accommodation. In Uganda, where 73% of healthcare funding comes from out-of-pocket contributions, procedures such as craniotomies and ventriculoperitoneal (VP) shunts remain unaffordable for most, consuming over 50% of a family’s annual income.

#### Geographical and societal barriers to neurosurgical care

Neurosurgical services are primarily available in urban centers, forcing rural patients to travel long distances for care, with the average distance in Ethiopia being approximately 144 km. Limited public transportation and the absence of prehospital emergency services further restrict timely access to life-saving neurosurgical interventions.

Social stigma, fear of surgery, and misconceptions about neurological conditions discourage patients from seeking neurosurgical care, leading many to rely on traditional healers. Political instability in countries like Sudan and Mali exacerbates the crisis, as conflicts disrupt healthcare services and contribute to the migration of skilled neurosurgeons to safer regions.

### Strengthening facilitators for neurosurgical care access in LMICs of Africa

#### International collaboration and partnerships

International partnerships have been vital in expanding neurosurgical capacity, with initiatives such as “Madaktari Africa,” the “Tanzania Neurosurgery Project,” and the “Duke East Africa Neurosurgery Program” improving training and service delivery. The WFNS Rabat Training Centre in Morocco has also significantly contributed by training over 50 neurosurgical residents and donating essential medical equipment.

#### Strengthening research in African LMICs

Collaborative research initiatives like the International Neurosurgical Twinning Modeled for Africa (INTIMA) and the Weill Cornell Tanzania Project have enhanced research capacity in neurosurgery, contributing significantly to hydrocephalus research and fostering continued support for research collaborations across Africa by institutions such as the Barrow Neurological Institute.

#### Telemedicine and telesurgery in African LMICs

Telemedicine has emerged as a pivotal tool in enhancing neurosurgical care across Africa, particularly by improving access, diagnostic turnaround times, and continuity of care. Notably, teleradiology and mobile health platforms have enabled the interpretation of over 58 000 imaging cases across 8 African countries, achieving an average turnaround time of just 2.46 hours. In Ethiopia, the adoption of telemedicine contributed to a 71% reduction in radiology wait times. Despite these promising outcomes, the long-term sustainability of telemedicine remains challenged by infrastructural limitations, including inconsistent internet connectivity, workforce shortages, and a lack of system interoperability. The COVID-19 pandemic underscored both the critical utility and inherent vulnerabilities of telemedicine systems. Continued progress will require strategic investment in digital infrastructure, targeted workforce training, and robust policy frameworks to ensure scalability and integration into national health systems.

The results of this study are summarized in Supplementary Digital Content Table 1.0, available at: http://links.lww.com/MS9/A926.

## Discussion and implications

### Barriers to accessing neurosurgical care in LMICs of Africa

#### Workforce deficit

A significant barrier to accessing high-quality neurosurgical care in LMICs across Africa is the critical shortage of neurosurgical professionals^[15]^. The continent currently faces an estimated 1.8 million unresolved neurological cases annually, necessitating nearly a 17-fold increase in the neurosurgical workforce to meet this demand^[3]^. The WHO recommends a ratio of 1 neurosurgeon per 200 000 individuals; however, in several African nations, this ratio can be as low as 1 neurosurgeon for every 687 740 people^[7]^. This shortage is exacerbated by the uneven geographic distribution of neurosurgeons, with most concentrated in urban centers and high-income countries, leaving rural areas and low-income countries largely underserved^[15]^. This deficiency directly impacts patient outcomes and the timeliness of care. In Uganda, where neurosurgical coverage is extremely limited, delays in managing emergency conditions, such as TBIs, often exceed the critical surgical window. A study found that delays of more than 4 hours in hospital arrival and failure to receive surgery were significantly associated with increased in-hospital mortality among TBI patients^[16]^. Similarly, in Ethiopia, wait times for treatable conditions like hydrocephalus and brain tumors can extend for weeks, allowing preventable complications to develop, worsening prognosis, or rendering patients inoperable^[17]^. Neurosurgical care requires a collaborative team approach, including anesthetists, nurses, and radiologists; therefore, sustained efforts to implement and support multidisciplinary teams are critical for effective neurosurgical care^[18]^. According to the Lancet Commission on Global Surgery, there is an annual need for an additional 143 million surgeries in LMICs, including neurosurgical procedures; yet, the existing surgical workforce in Africa is particularly inadequate to meet this growing demand^[19]^. As of 2020, the distribution of the neurosurgical workforce, specifically neurosurgeons, and workforce density varies across various regions of Africa^[7]^.

This is outlined in Supplementary Digital Content Table 2.0, available at: http://links.lww.com/MS9/A926.

#### Barriers to neurosurgical education and training programs

Institutions in Africa that offer neurosurgical residency programs play a crucial role in expanding access to neurosurgical services. However, despite Africa having 54 countries, only 32 currently provide such training programs^[20]^. LMICs in Africa that do offer neurosurgical training face significant challenges, including limited financial resources, poor infrastructure, and a lack of standardized training curricula. These factors have resulted in inconsistencies in resident recruitment and training durations, creating an unfavorable environment for developing neurosurgical talent^[20]^. Furthermore, while interest in neurosurgical training is growing among students, existing programs lack the capacity and infrastructure to accommodate all applicants, necessitating partnerships with international residency programs^[21]^. The training capacity in these 32 countries could serve as a strategic foundation for scaling up enrollment, harmonizing standards, and fostering cross-national collaboration across the continent. Despite these structural limitations, some neurosurgical training programs in Africa have successfully produced competent, internationally recognized neurosurgeons. For instance, alumni of the WFNS Rabat Training Centre in Morocco and the CURE Children’s Hospital of Uganda have assumed leadership roles and contributed to international research^[22,23]^. External evaluations of programs like the neurosurgical initiative at Mulago Hospital in Uganda have demonstrated strong surgical competence and growing operative independence among locally trained neurosurgeons^[24]^. This demonstrates that, even in constrained settings, well-supported training programs can produce high-quality specialists. The COVID-19 pandemic further disrupted neurosurgical training in Africa, particularly in LMICs^[25]^. While 98.5% of neurosurgical training programs in high-income countries in North America transitioned to online platforms, only 43.9% of African respondents reported similar shifts to digital learning, highlighting the disparity between LMICs and high-income countries^[25]^. Additionally, during the pandemic, African neurosurgical residents faced examination delays, with 19.5% experiencing cancellations and 57.7% encountering postponements, emphasizing the inadequacies in training facilities across LMICs in Africa^[26]^.

#### Resource and infrastructure deficits

Neurosurgical care in African LMICs faces significant infrastructure and resource limitations. Access to essential equipment such as operating microscopes, intraoperative fluoroscopy, and radiofrequency generators remains scarce. Countries like Togo and Guinea report critical shortages of ICU beds; even where beds exist, the number of ICU units is often inadequate^[8]^. Staff shortages, particularly in anesthesia and nursing, contribute to delayed procedures^[8]^. Laboratory constraints also undermine care, as illustrated by a case in which a 16-month-old with a posterior fossa tumor developed a multidrug-resistant infection due to unavailable reagents and cerebrospinal fluid collection tubes^[27]^. Additionally, clinicians often manage limited resources without formal administrative training, which adds to their burden and limits optimal equipment use^[27]^.

#### Financial constraints and out-of-pocket costs

Financial barriers and out-of-pocket expenses pose significant challenges to accessing neurosurgical care in African LMICs. In Eastern Africa, families may spend up to $539.44, approximately 132% of their annual income, on neurosurgical treatment, with indirect costs like transportation and accommodation adding another $79.37 (19.7% of income)^[28]^. Although neuroimaging services like CT scans are relatively cheaper than in high-income countries, they remain unaffordable for 80–90% of the population, with little price variation between public and private facilities^[29]^. In Uganda, out-of-pocket payments account for 73% of healthcare funding, with procedures like craniotomies ($230.68) and VP shunts ($449.56) costing up to half of a family’s annual income, which averages $533^[30,31]^. Overhead costs for these procedures also range from 29% to 41%, straining both patients and national health systems^[32]^. These financial barriers to accessing neurosurgical care in LMICs across Africa can be summarized in Supplementary Digital Content Table 3.0, available at: http://links.lww.com/MS9/A926.

#### Geographical barriers to neurosurgical care

Geographical challenges significantly impact access to neurosurgical care across African LMICs, as neurosurgeons and their treatment facilities are largely concentrated in urban areas. This concentration forces patients from rural regions to travel considerable distances to receive care^[8]^. In Ethiopia, for example, the average distance a patient travels to reach a surgical center is approximately 144 kilometres^[33]^. There is also a trend of neurosurgical trainees migrating to private urban clinics or even neighboring countries, further reducing the availability of care in remote areas^[8]^. Consequently, many rural patients turn to informal providers such as traditional healers, who are more accessible, have a strong community reputation, and are affordable^[27]^.

Even in urban areas where facilities are present, challenges persist, such as limited access to private transportation, a lack of free prehospital emergency care, and inconsistent public transportation depending on the region^[27]^. The Lancet Commission on Global Surgery recommends that surgical intervention should be accessible within 2 hours of need; yet, only 25.3% of the population in sub-Saharan Africa meets this standard^[2,34]^. Emerging evidence suggests that long travel distances not only delay treatment but also worsen clinical outcomes. For instance, a prospective registry of TBI patients at Mulago National Referral Hospital in Uganda found that those who arrived more than 4 hours after symptom onset had significantly higher mortality rates, delays often attributed to the lack of timely neurosurgical access in rural areas^[16]^.

#### Cultural, political, and societal barriers

Cultural and societal factors can impede access to neurosurgical care by influencing patients’ health-seeking behaviors. These factors may include social stigma, fear, and mistrust of the healthcare system^[35]^. One study revealed that many patients in LMICs in sub-Saharan Africa avoid seeking treatment for neurological conditions due to cancer-related stigma and widespread misconceptions, often opting for traditional or alternative therapies instead^[36]^. Furthermore, a lack of awareness and understanding of conditions like brain tumors among patients presents another challenge to accessing neurosurgical services^[37]^.

Political instability and ongoing conflicts in countries such as Sudan and Mali further complicate the delivery of neurosurgical care. These geopolitical disturbances disrupt medical services and lead to widespread socio-economic consequences that negatively impact healthcare systems^[38]^. A significant result of this instability is the migration of skilled healthcare workers, including neurosurgeons, to safer regions in search of better career opportunities, exacerbating the shortage of medical professionals and limiting access to critical care for patients^[38]^.

These barriers to accessing neurosurgical care in middle-income countries across Africa can be summarized in Supplementary Digital Content Table 4.0, available at: http://links.lww.com/MS9/A926.

### Strengthening facilitators for neurosurgical care access in LMICs of Africa

#### International collaboration and partnerships

International collaboration and partnerships have emerged as key strategies to address access to neurosurgical care in LMICs across Africa. However, coverage remains limited, with most collaborations being short-term, concentrated in urban centers, and insufficiently integrated into local health systems^[21,24]^, given the high unmet surgical need^[1]^. Over the past 15 years, several international partnerships, such as Madaktari Africa, the Tanzania Neurosurgery Project by Weill Cornell Medical College, and the Duke East Africa Neurosurgery Program led by Dr Michael Haglund, have significantly contributed to neurosurgical capacity building in East Africa^[1]^. These initiatives have supported the training of local surgeons, the organization of fellowships with teleconferencing, and the training of neurosurgeons from neighboring countries. Notably, the WFNS Rabat Training Centre in Morocco has trained over 50 neurosurgical residents from sub-Saharan Africa, supported through equipment donations and long-term mentorship^[22]^.

Evidence indicates that these partnerships have a demonstrable and sustainable impact. For instance, the Madaktari Africa “train-forward” model in northern Tanzania increased the number of neurosurgical procedures from 18 in 2005 to approximately 92 annually by 2010, with local providers performing 86% of cases independently and a significant decrease in postoperative complications^[39]^. Similarly, alumni of the WFNS Rabat Training Centre have returned to practice in 12 sub-Saharan African countries, collectively serving a population of 267 million and dramatically improving neurosurgeon-to-population ratios from 1 per 6 million to 1 per approximately 2.6 million inhabitants^[40]^. A review of East and West African neurosurgical education collaborations confirms that these programs have progressively increased capacity across the continent, although further infrastructure investment is still required to sustain momentum^[21]^.

Nonetheless, these collaborations face notable limitations. The Duke East Africa Neurosurgery Project confronted significant resource constraints, cultural and cross-team coordination challenges, and difficulty retaining trained personnel in-country, key barriers to maintaining consistent training and service delivery^[41]^. Other obstacles include reliance on visiting faculty, interruptions of programs during funding lapses, equipment maintenance issues, and weak post-training mentorship – issues highlighted in regional neurosurgical education reviews^[1]^. Future efforts must prioritize local ownership, program continuity, and integration into national health systems to maximize sustainability.

#### Strengthening research in African LMICs

Research is vital for enhancing neurosurgical care in African LMICs, with collaborative projects led by international partners such as the INTIMA. This initiative promotes collaboration between Swedish and African neurosurgeons to advance research^[42]^. The Weill Cornell Tanzania Project has also created a significant resource for neurosurgical research by establishing a patient registry and database for conditions such as spine trauma, TBI, and hydrocephalus, thereby laying a foundation for further research efforts^[23]^. Another key contributor to neurosurgical research in Africa is CURE International, a leader in hydrocephalus research, particularly through the reintroduction of choroid plexus cauterization combined with endoscopic third ventriculostomy^[23]^. Additionally, the Barrow Neurological Institute in the United States has been active in fostering research collaborations across African LMICs^[3]^. Despite these advancements, challenges such as fragmented research systems, limited funding, and a lack of practical research experience continue to hinder the expansion of research capacity in African LMICs^[43]^.

#### Telemedicine and telesurgery in African LMICs

In the absence of advanced infrastructure, telemedicine has emerged as a promising complementary tool for African neurosurgical care. Although its adoption has been uneven, especially during the COVID-19 pandemic, studies have shown improvements in access, speed, and quality of care through teleconsultation, teleradiology, and remote follow-up services in several Sub-Saharan African countries. However, telemedicine systems in many SSA regions remain unsustainable due to a heavy reliance on short-term pilot projects and poor integration into national health systems^[44]^. Similar teleradiology initiatives in countries like Nigeria have proven feasible in supporting rural diagnostics, particularly through mobile platforms designed for triage and follow-up care, not as surgical substitutes, but as capacity-building tools^[45]^.

Sustainability and long-term impact are critical considerations. A multicountry retrospective study covering 8 African countries found that over 58 000 imaging studies were successfully interpreted via teleradiology platforms between 2017 and 2022, with an average turnaround time of 2.46 hours, highlighting both efficiency and scalability^[46]^. In Ethiopia, a quasi-experimental evaluation of a web-based teleradiology platform in Northwest public hospitals demonstrated a 71% reduction in radiology waiting time (from a median of 43.5 to 4.6 hours) and an 11% increase in patient satisfaction, illustrating clear, quantifiable gains in both efficiency and service quality^[47]^. Similarly, a 2024 systematic review across South Africa, Kenya, and Nigeria noted that while telemedicine platforms have been successful in chronic disease management and education, broader implementation in surgical disciplines still requires consistent investment in digital infrastructure, policy support, and provider training^[48]^.

The COVID-19 pandemic significantly disrupted neurosurgical care, impacting both patient management and surgical training globally^[49]^. Lockdowns and resource reallocation caused delays in elective neurosurgical procedures, reducing patient access to timely interventions^[49]^. In LMICs, where neurosurgical services were already limited, these disruptions exacerbated existing barriers to care^[50]^. Training was also severely hampered, as restrictions on in-person learning and surgical exposure limited hands-on experience for neurosurgical trainees^[51]^. While in-person visits remain the ideal option, telemedicine offers critical insights, especially in emergencies^[49]^. It also serves as a promising tool for training healthcare professionals in neurosurgery^[51]^. However, several challenges hinder its widespread adoption. Poor internet connectivity and frequent power outages in LMICs disrupt virtual consultations, limiting the reliability of telemedicine^[50,52]^. Additionally, low technological literacy among healthcare providers and patients reduces effective engagement with telemedicine platforms^[50,51]^. Ethical concerns regarding data privacy and a weak cybersecurity infrastructure further complicate implementation^[50,52]^.

Furthermore, the Weill Cornell Tanzania project has successfully utilized mobile-based platforms for postoperative follow-ups in hydrocephalus cases, enhancing continuity of care in remote settings^[53]^. These digital innovations reflect the double-edged sword of artificial intelligence in healthcare, providing significant opportunities to expand access while simultaneously risking greater inequities in low-resource settings if not supported by localized infrastructure. For example, AI platforms like AlphaFold have revolutionized drug discovery in well-resourced environments^[54]^, while large language models have deepened disparities in areas with limited digital capacity^[55]^. The success of telemedicine and remote training in neurosurgery will ultimately depend on coordinated investments in digital readiness and equitable system strengthening.

These facilitators for improving access to neurosurgical care across LMICs are summarized in Supplementary Digital Content Table 5.0, available at: http://links.lww.com/MS9/A926.

## Advantages and disadvantages of this review

This narrative review offers a broad and flexible synthesis of literature exploring neurosurgical care in African LMICs, which is particularly valuable in fields with limited or heterogeneous data. By integrating diverse sources, from academic publications to regional initiatives, this approach provides a holistic view of systemic challenges and emerging facilitators, making it relevant for policymakers, clinicians, and global health actors. The narrative format also aids in identifying thematic gaps and generating context-specific hypotheses, which may guide future research or inform strategic planning. However, this flexibility comes with inherent drawbacks: narrative reviews are subject to selection bias due to the lack of strict inclusion protocols, and they do not include formal quality appraisal or meta-analysis. This limits reproducibility and may affect the objectivity and generalizability of findings. Nonetheless, in regions like sub-Saharan Africa, where literature is sparse, fragmented, and context-sensitive, a narrative review remains a pragmatic tool for synthesizing existing knowledge and advocating for structural reforms.

## Limitations

While this study provides valuable insights into the barriers and facilitators of neurosurgical care in LMICs across Africa, several limitations must be acknowledged. The reliance on existing literature introduces potential publication bias, as studies with positive findings are more likely to be published, which may lead to an incomplete representation of challenges and opportunities. Additionally, variations in healthcare systems, socioeconomic factors, and geopolitical stability across African LMICs may limit the generalizability of the findings, as healthcare infrastructure and policies differ significantly. Data availability and quality also pose challenges, with many LMICs lacking comprehensive neurosurgical registries and robust reporting systems, impacting the accuracy of analyses. Furthermore, the study primarily relies on secondary data sources, which may not fully capture the perspectives of key stakeholders such as neurosurgeons, policymakers, and patients; incorporating qualitative methods could provide richer insights. Lastly, language restrictions and database selection may have influenced the scope of included literature, potentially overlooking relevant studies published in non-English languages.

Despite these limitations, this study contributes to the ongoing discourse on improving neurosurgical care in African LMICs, highlighting areas for future research and policy interventions to strengthen healthcare systems.

## Conclusion

The key takeaway from this review is that effective neurosurgical care in African LMICs depends on regionally tailored strategies, strengthened workforce training, and multi-stakeholder collaboration. Improving services requires not only more resources but also coordinated investment in training, regional centers of excellence, and innovations like telemedicine. The severe workforce shortage, combined with geographic and financial barriers, makes it urgent for governments and global actors to prioritize neurosurgery within essential public health infrastructure.

To address these barriers, governments should allocate national funding for neurosurgical training programs, including scholarships and incentives for rural deployment. They must also procure and maintain essential neurosurgical equipment (e.g., imaging, ICU beds, microscopes) and integrate neurosurgery into national health insurance schemes to reduce out-of-pocket costs. The African Union should establish regional neurosurgical training and care hubs under its Health Strategy to ensure decentralized service delivery and workforce development. International organizations such as the WHO and WFNS can provide technical support for telemedicine platforms, help develop standardized surgical curricula, and offer seed grants for neurosurgical infrastructure. NGOs and academic partners should commit to capacity-building agreements with African institutions, focusing on mentor exchange, equipment donations, and digital training modules. Strengthening continental networks like PAANS and enabling the Africa CDC to coordinate neurosurgical data and training will ensure long-term sustainability.

## Data Availability

All data pertaining to manuscript are available upon request from the Principal Investigator.

## Research registration unique identifying number (UIN)

This manuscript does not involve any human participants.
